# Effects of food limitation on growth, body condition and metabolic rates of non-native blue catfish

**DOI:** 10.1093/conphys/coaa129

**Published:** 2021-01-08

**Authors:** Vaskar Nepal, Mary C Fabrizio, Richard W Brill

**Affiliations:** Department of Fisheries Science, Virginia Institute of Marine Science, William & Mary, P.O. Box 1346, Gloucester Point, VA 23062, USA; Department of Fisheries Science, Virginia Institute of Marine Science, William & Mary, P.O. Box 1346, Gloucester Point, VA 23062, USA

**Keywords:** Blue catfish, Chesapeake Bay, food limitation, invasive species, metabolic rate

## Abstract

Establishment and range expansion of non-native species in novel habitats depend on their energetic requirements and food availability. Knowledge of growth and metabolic rates of non-native fishes at various food levels is particularly critical to inform models that assess their invasion potential. We compared growth rates, body condition and metabolic rates of juvenile blue catfish (*Ictalurus furcatus*), an invasive species in many lakes, coastal rivers and estuaries throughout the Eastern USA, at three ration levels: *ad libitum* (3.5% of fish body mass/d), two-third *ad libitum* and one-third *ad libitum*. All fish survived the entire duration of the experiment (4 months) regardless of ration level. Blue catfish exhibited routine metabolic rates similar to those of other benthic fishes but below the more active species. Mean growth rates were lower at reduced ration levels, but we found no evidence of ration size effect on body condition or metabolic rates. Blue catfish therefore appear to have mechanisms that enable them to survive low rates of food intake for long periods, indicating the potential of this invasive species to become established in habitats with low prey availability.

## Teaser

In a laboratory experiment, invasive blue catfish survived well, grew and maintained a good body condition at food levels as low as one-third of the maximum. Blue catfish had low metabolic rates, suggesting that this species has a potential to invade many low-food habitats in the eastern USA.

## Introduction

The number of established non-native species has increased worldwide in recent centuries (Seebens *et al.*, 2017), and this increase is expected to continue (Essl *et al.*, 2011). A portion of these non-native species become invasive with devastating ecological and economic impacts (Lockwood *et al.*, 2013). Establishment success and impacts of such species depend, in part, on their physiological abilities and limitations (Lennox *et al.*, 2015; Meyerson *et al.*, 2019). In particular, the energetic requirements and responses of non-native fish to variable food regimes directly influence their role and trophic impacts in novel ecosystems.

Food is a driving force governing the growth and metabolism of fishes (Brett and Groves, 1979). A mismatch between the demand for and the availability of food resources may, therefore, impede the establishment of a non-native species in novel habitats. In a community where multiple species compete for limited resources, the resource-ratio hypothesis predicts that species with the lowest resource requirements (i.e. R^*^) will outcompete other species when resources are drawn below the levels that other species can tolerate (Tilman, 1982). For example, in mesocosm studies, invasive bighead carp (*Hypophthalmichthys molitrix*) outcompetes native paddlefish (*Polyodon spathula*) through low metabolic demands and superior feeding efficiency (Schrank *et al.*, 2003).

The characterization of non-native fishes’ consumptive demands and responses to limited food resources can inform population and bioenergetics models that can predict their potential trophic impacts and population dynamics in novel environments. For example, Cooke and Hill (2010) developed a bioenergetics model for invasive Asian carp (*Hypophthalmichthys* spp.) to identify both the environmental conditions and specific basins in the Laurentian Great Lakes that may be susceptible to invasion due to the low metabolism and low consumptive demands of the fish. Similarly, a full life-cycle bioenergetics model for the purple mauve stinger (*Pelagia noctiluca*), an invasive holoplanktonic cnidarian in the Mediterranean Sea, revealed that individuals could mature and reproduce even at ingestion levels as low as 14% of maximum (Augustine *et al.*, 2014). Bioenergetics models such as these, however, require information on growth, body condition and metabolic rates of the species at specific food densities. Bioenergetics models can then provide insights on growth and feeding rates observed in the wild (Kooijman, 2010). An ability to grow and maintain a good body condition in restricted food environments would be beneficial for an invading organism.

The estimates of metabolic rates of an invasive species can also inform growth dynamics (van Poorten and Walters, 2016), elucidate ecological traits (Killen *et al.*, 2016) and support the development of bioenergetics models for the species (Cooke and Hill, 2010; Kooijman, 2010). Species with low maintenance costs, as measured by standard metabolic rate (SMR) in fish, are more likely to succeed under harsh, patchy or unpredictable environmental conditions that invading species may experience in novel ecosystems (Tilman 1982; Reid *et al.*, 2012). Additionally, a high maximum capacity to mobilize energy, as measured by maximum metabolic rate (MMR), may increase foraging rates and behavioural dominance (Metcalfe *et al.*, 2016). A change in the relative ability of an organism to expend energy beyond that required for homeostasis, as measured by the factorial scope (FS = MMR/SMR) under restricted food conditions provides a holistic indicator of fish health. Bringing these ideas together, the ‘compensation hypothesis’ predicts that a lower SMR allows for the allocation of more energy to growth and reproduction (Deerenberg *et al.*, 1998), two processes that facilitate invasion. Finally, the ability to decrease maintenance costs (and hence, SMR) during restricted food conditions (observed in many fish species; e.g. Van Leeuwen *et al.*, 2012; Auer *et al.*, 2016; Liu and Fu, 2017) may offer a competitive advantage for the colonization of, and the establishment in, novel habitats.

To help predict the establishment success and impacts of invasive blue catfish (*Ictalurus furcatus*), we quantified the effects of food limitation on their growth, condition and metabolic rates. A primarily freshwater fish native to large rivers in the Midwestern USA, blue catfish has been introduced throughout North America to promote recreational fisheries (Fuller and Neilson, 2020). During the 1970s and 1980s, blue catfish were stocked in the tidal freshwater regions of three rivers in Virginia but have since become established in all major tributaries of the Chesapeake Bay (Fabrizio *et al.*, n.d.; Schloesser *et al.*, 2011). Owing to the range expansion, increase in relative density and abundance and potential negative effects on native fish and shellfish resources, blue catfish are now considered an invasive species in the region (Fabrizio *et al.*, 2018; Nepal and Fabrizio, 2019, 2020a; Schmitt *et al.*, 2019). Blue catfish are also of concern in coastal rivers and lakes throughout the Eastern USA, including Delaware, North Carolina and Georgia (Homer and Jennings, 2011; Bonvechio *et al.*, 2012). As such, resource managers are now developing policies and regulations to limit their dispersal, population size and potential trophic impacts (Fabrizio *et al.*, n.d.).

The energetics of wild blue catfish, although important in understanding their invasion and establishment success, is currently not known. Schmitt (2018) estimated the maximum consumption rates of wild blue catfish from the Chesapeake Bay region to be ~9% body weight per day, but the impacts of reduced ration on blue catfish biology have not been studied. If blue catfish have lower food requirements than native species, and if they can persist under low food levels, then they may become established in low-food environments throughout Eastern USA, where they may negatively impact the diversity, density and health of native species through exploitative competition (Hart and Marshall, 2012). If, however, blue catfish have high energetic requirements, they may be successful in a novel ecosystem if they are able to efficiently seize resources from established native residents via interference competition (Hart and Marshall, 2012). We also do not know if blue catfish are able to lower their metabolic demands during prolonged periods of food limitation to allow better survival during such situations.

The main objective of our study was to understand the food and energetic requirements of blue catfish to (i) characterize the role of this invasive species in non-native habitats throughout the Eastern USA and (ii) inform bioenergetics and population models that examine the future distribution, dynamics and impacts of this species. To do this, we monitored the body length and condition and three metabolic rate indices [MMR, SMR and FS] of individuals subjected to one of three ration sizes for 4 months.

## Materials and Methods

All animal capture, handling and experimental procedures were approved by the William & Mary Institutional Animal Care and Use Committee (protocols IACUC-2015-06-30-10 455-mcfabr and IACUC-2017-05-22-12 111-tdtuck) and followed all applicable US guidelines.

### Experimental system

We assessed the effects of reduced ration size on growth, body condition and metabolic rates of juvenile blue catfish because growth is fastest in young fish. We captured fish using a 9.14-m otter trawl from the James River subestuary following sampling protocols of the Virginia Institute Marine Science (VIMS) Juvenile Fish Trawl Survey. Fish were transported to the VIMS Seawater Research Laboratory, anaesthetized using clove oil (50-mg/L concentration), weighed and tagged with unique 8.4-mm passive integrated transponder tags using a sterile syringe injector. Fish were held in 500-L cylindrical tanks at 20°C and 1.5 psu (practical salinity units) for at least 2 weeks prior to use in the experiment. During this time, they were fed commercial slow-sinking catfish pellets *ad libitum* three times per week. The 3-mm pellets were fishmeal-based and contained 40% protein and 10% fat (Zeigler Bros, Inc.).

We used a randomized nested experimental design with five blocks and three treatment levels. Blocks were represented by five recirculating aquaculture systems (RASs), each of which contained three 270-L cylindrical aquaria supplied with mechanical and biological filtration devices. The five RASs ensured uniformity of temperature conditions among the three aquaria within each RAS, and among the RASs. Individuals were sorted by fork length (FL) and randomly assigned to one of the five RASs (i.e. one of the five blocks), such that each aquarium within an RAS received two fish of similar size. We held two fish in each aquarium because feeding declined considerably when only one individual was present in an aquarium. We did not know the sex of the fish at the start of the experiment. Each aquarium within an RAS was randomly assigned to one of three ration size treatment levels: *ad libitum*, two-third of *ad libitum* (‘two-thirds’ hereafter) and one-third of *ad libitum* (‘one-third’ hereafter).

To estimate the *ad libitum* ration size, we added fixed amounts of food (either 2.0, 2.5, 3.0, 3.5, 4.0, 4.5, 5.0, 5.5 or 6.0% of the total fish biomass) to each aquarium. These pre-trials were conducted three times with 48-hour intervals between trials; fish were starved for 34 hours between each trial. The greatest mean ration size that was fully consumed overnight (14 hours) by fish (3.5% of total fish biomass) was chosen as the *ad libitum* ration size. Ration size based on the proportion of fish weight results in a fish with lower body condition (lower body mass for its length) receiving smaller ration than another fish of the same length but with higher body condition, potentially resulting in further loss of body mass over time by the lower-conditioned fish. To avoid this, we estimated ration size for each fish using the relationship between ration and fish length, rather than fish weight. We fit a linear regression between mass-based ration size (g) for each fish and the square of fork length (FL^2^) of the fish; we chose FL^2^ because bioenergetics theory suggests that consumption rates in fish are proportional to mass^2/3^ (Kooijman, 2010; van Poorten and Walters, 2016), which is equivalent to length^2^ for a fish growing isometrically. We used the linear regression model (ration = 0.0114 $\times$ FL^2^–1.7257; R^2^ = 0.85; *n* = 30) to calculate mean ration size (g) for each fish based on its length. The total allotted ration size for each pair of fish in a given aquarium was the sum of the estimated ration sizes for the two fish.

We adjusted ration size for each pair of fish using the above ration-FL^2^ relationship at 1-month intervals to account for fish growth. Fish assigned to *ad libitum* rations were fed daily and those assigned to one-third ration size were fed full rations every third day; fish in the two-thirds ration size were fed a full ration two consecutive days but not on the third day. Fish were fed between 4:30 and 8:00 PM, and excess food was siphoned from each aquarium the next morning. We monitored water quality (dO_2_, salinity, NH_3_, NO_3_^−^ and NO_2_^−^) twice per week and performed water changes as necessary to maintain water quality. All systems were maintained at 20°C (range 18.6–21.1${}^{\circ}$C) and 1.5 psu (range 1.3–1.7 psu) to prevent parasitic infestations that we commonly observed at lower salinities.

At the start of the experiment, we measured metabolic rates of each fish using intermittent-flow respirometry protocols as described below. Following the respirometry trial, fish were returned to their respective aquaria. We performed five respirometry trials on each individual with a 1-month interval between trials. To ensure that time intervals between two monthly measurements were similar for all individuals, we used individuals in the respirometry trials in the same order each month, with the order during the first month being random. The condition of the fish was calculated for each month as Fulton’s condition factor (*K*):


(1) \begin{equation*} K=\mathrm{100,000}\times W\times F{L}^{-3}, \end{equation*}

where *W* is the weight of the fish.

During the third measurement period, four fish died due to the malfunction of the respirometry system. These fish were replaced with newly collected individuals from the James River that were allowed to acclimate to laboratory conditions for 7 days prior to tagging and obtaining length and weight measurements. At the conclusion of the experiment (4 months), all fish were euthanized by immersion in an ice slurry and subsequently sexed by macroscopic examination of the gonads.

### Measurement of metabolic rates

We used intermittent-flow respirometry (Svendsen *et al.*, 2016b) to determine the SMR and MMR of blue catfish (*n* = 10 from each ration size, total = 30) at 20°C and 1.5 psu. Respirometry trials were conducted in 4-L cylindrical respirometry chambers (Loligo Systems, Viborg, Denmark). To ensure that chamber volumes were no more than 50 times the volume of the fish (as recommended by Svendsen *et al.*, 2016a), we reduced the volumes of the chambers by inserting sealed glass flasks of known volume into the chambers. The flasks did not hinder water circulation during measurement periods or during chamber flushing.

Metabolic rates were measured in four independent respirometry chambers (one fish per chamber), each submersed in temperature-controlled water baths bubbled with air to maintain normoxic conditions. We placed an opaque cover over the water bath and respirometry chamber to minimize visual disturbance. The oxygen saturation (%) of the water in the chamber was measured every second by a pre-calibrated FireSting fibre-optic oxygen metre (Pyro-science, Aachen, Germany), the sensor of which was inserted in the water circulation tubing. We converted oxygen saturation to oxygen concentration ($[{O}_2],$mg O_2_ L^−1^) using standard equations based on temperature, salinity and partial pressure. The mean oxygen consumption rate $(\Delta \dot{[{O}_2],}$ mg O_2_ L^−1^ h^−1^$)$ during each cycle was calculated as the slope of a linear regression of recorded oxygen concentrations against elapsed time and was subsequently converted to mass-specific oxygen consumption rates ($\dot{M}{O}_2$, mg O_2_ kg^−1^ h^−1^) using the following relationship:


(2) \begin{equation*} \dot{M}{O}_2=\Delta \dot{\left[{O}_2\right]}\times V\times{W}^{-1}, \end{equation*}

where *V* is the volume of the respirometry chamber (L) corrected for fish and flask volume and *W* is the weight of the fish (kg) at the time of the respirometry trial. Each trial was automated by controlling the pumps using the open-source software program AquaResp (www.AquaResp.com). For each trial, we used flush and wait and measure for 3, 1 and 7 min, respectively.

For each respirometry chamber, background respiration due to bacteria (i.e. the rate of oxygen depletion in the respirometry chamber without a fish) was measured at the start and end of each week. Data were collected over twenty-four 1-hour measurement periods and ranged between 0% and 17.3% of the fish oxygen consumption, with a median of 0.3% and 95th percentile of 8.3%. We estimated background respiration rate throughout the week assuming a linear relationship between background respiration measurements taken at the start and the end of the week. The oxygen consumption rate for each fish was subsequently corrected for background respiration by subtracting the estimated background respiration on the day of the trial (Svendsen *et al.*, 2016b). We did not use parallel chambers to measure background respiration because preliminary trials showed that most of the variation in background respiration was chamber-specific but highly repeatable for each chamber. To minimize background respiration, all chambers, tubing and flasks were cleaned in a 10% bleach solution at the start of each week.

We starved the fish for a minimum of 40 hours before the respirometry trials to ensure post-absorptive state. Fish were exercised to exhaustion (the point at which they no longer elicited an escape response to handling) in a water flume and subjected to a brief period (~1 min) of air exposure. This protocol is suited for eliciting MMR in benthic fish species that do not regularly exhibit prolonged swimming (Killen *et al.*, 2017). Fish length and weight were recorded immediately prior to the fish being placed into the respirometry chamber, after which they remained for 21–29 hours. We determined the MMR of each fish as the highest recorded $\dot{M}{O}_2$ and the SMR as the 20th percentile of $\dot{M}{O}_2$, excluding the first 10 hours after introduction (Chabot *et al.*, 2016). FS was calculated as the ratio of MMR to SMR of each fish. Absolute scope, calculated as the difference between MMR and SMR, was not used in subsequent analyses because we found a strong correlation with MMR (correlation coefficient *r* = 0.98; *P* < 0.001; see below).

### Statistical analysis

We first assessed the correlations among the response variables (FL, K, SMR, MMR and FS) using pairwise Pearson’s correlations, *r*. We considered two variables with *r* less than 0.3 to be weakly correlated and those with *r* greater than 0.6 to be strongly correlated. Strong correlation between two response variables suggests mutual dependence and correlated errors, thus requiring a joint modeling approach. Correlations among the variables were generally weak, however, allowing us to model and examine each response variable independently.

We assessed the effects of ration size and sex of fish on body length, body condition and metabolic rates of blue catfish using separate mixed-effects repeated measures models for each response variable. The model for FL took the following form:


(3) \begin{equation*} F{L}_{ijkl}=\mu +{S}_i+{R}_j+D+{B}_k+{R}_j\times D+{I}_{k(l)}+{\varepsilon}_{ijkl}, \end{equation*}

where *μ* is the overall mean response, *S_i_* the effect of sex *i*, *R_j_* the effect of ration size *j*, *D* the number of days since the start of the experiment, *B_k_* the baseline or initial FL of fish *k* (i.e. FL observed on the start date of the experiment), *R_j_ × D* the interaction between ration size and the number of days since the start of the experiment, *I*_*k*(*l*)_ the random effect of fish *k* nested within RAS *l* and *ε_ijkl_* the unexplained random variation.

We included fish weight at the time of monthly measurement as a covariate in mixed models for K, SMR, MMR and FS because body mass affects Fulton’s body condition (Froese, 2006) as well as metabolic rates of fish (Schmidt-Nielsen, 1984; Killen *et al.*, 2016):


(4) \begin{equation*} {Y}_{ijkl}=\mu +{S}_i+{R}_j+D+{B}_k+{R}_j\times D+{I}_{k(l)}+{W}_k+{\varepsilon}_{ijkl}, \end{equation*}

where *Y_ijkl_* is the response and *W_k_* the effect of wet weight of fish *k*. Other symbols are as previously defined. We note that mass-specific metabolic rate declines exponentially with fish weight (Killen *et al.*, 2016); this decline is well approximated by a linear relationship over a narrow range of fish weights, as was the case in our study. For each model, our primary interest was in the ration size-by-time interaction *R_j_ × D*, which, if significant, would indicate variable effects of time on the observed response (length, condition or metabolic rates) depending on ration size. We did not include other interaction terms because preliminary graphical analysis indicated no interactions.

We assessed various covariance structures to account for potential autocorrelations in repeated measurements of each response. Specifically, we considered unstructured, first-order autoregressive (ar1), Toeplitz and spatial power covariance structures to model autocorrelations in response variables over time (Stroup *et al.*, 2018). We note that autoregressive and Toeplitz covariance structures assume equally spaced time intervals between repeated measurements (Stroup *et al.*, 2018), even though in our experiment, the time intervals among individual measurements ranged from 28 to 35 days. By fitting these covariance structures, we therefore assumed that intervals of 28–35 days were equivalent. Covariance structures with heterogeneous variances, such as heterogeneous autoregressive and heterogeneous Toeplitz were also considered (Stroup *et al.*, 2018). We fitted models with these alternative covariance structures using restricted maximum likelihood (Stroup *et al.*, 2018) and compared them using Akaike information criterion corrected for small sample sizes (AICc) (Burnham and Anderson, 2002). For each response variable, we refitted the most parsimonious model of the covariance structure (defined as the one with the lowest AICc value) with maximum likelihood to obtain unbiased parameter estimates for the fixed effects (Stroup *et al.*, 2018).

We mean-centred some of the predictor variables to aid computations and suppressed the intercept to aid model interpretation. Assumptions of homogeneity of variance and normality were assessed using diagnostic plots. All statistical analyses were conducted in SAS version 9.4 (SAS Institute, Cary, NC) following procedures outlined in Stroup *et al.* (2018). Statistical significance was set at *α* = 0.05.

## Results

**Figure 1:**
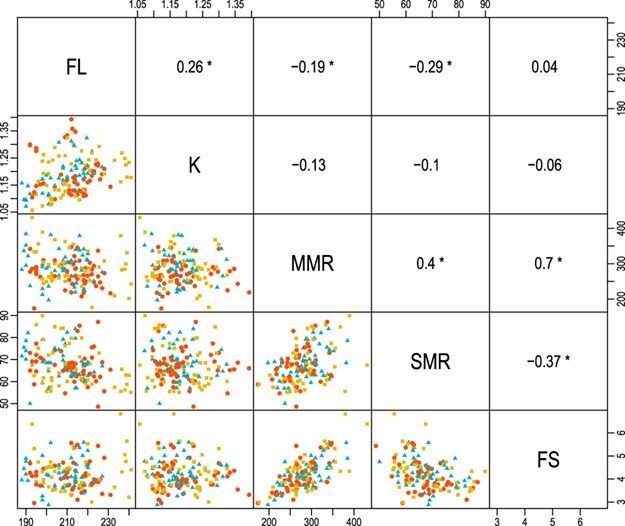
Scatterplot matrix of FL, K, SMR, MMR and FS of juvenile blue catfish under *ad libitum* (▲), two-thirds of *ad libitum* (●) and one-third of *ad libitum* (■) ration size. Upper panels show the pairwise Pearson’s correlation coefficients, which are significant at α = 0.05 if accompanied by an asterisk (*). See text for details on units of each variable.

Mean FL was 207 mm (range 188–241 mm) and mean wet weight was 114 g (range 73.5–170.5 g) at the beginning of the experiment. We found a weak positive correlation between FL and K (*r* = 0.26; *P* = 0.001; Fig. 1). MMR and SMR were also positively correlated with each other (*r* = 0.40; *P* < 0.001). FS had a relatively strong positive correlation with MMR (*r* = 0.70; *P* < 0.001) but a weak negative correlation with SMR (*r* = −0.37; *P* < 0.001; Fig. 1). SMR and MMR were weakly correlated with FL (|*r*| ≤ 0.29; *P* < 0.05). None of the metabolic rate indices were correlated with body condition (|*r*| ≤ 0.13; *P* > 0.05).

**Figure 2:**
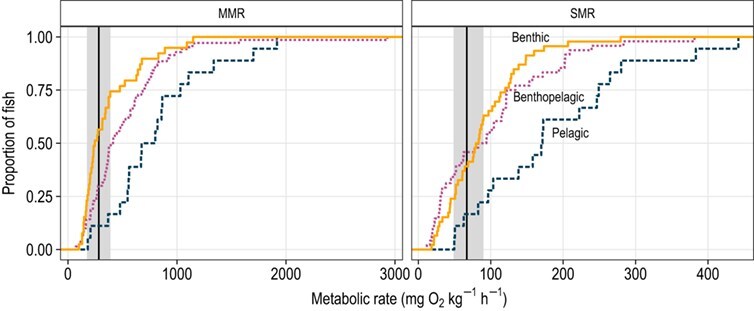
Comparison of the MMR and SMR of juvenile blue catfish with those of other benthic (solid lines), benthopelagic (dotted lines) and pelagic fishes (dashed lines). Black vertical lines represent the mean and grey rectangles represent the ranges of MMR and SMR values for blue catfish. All data are standardized to 1 kg and 20°C. Data for MMR (*n* = 121) and SMR (*n* = 112) were obtained from the supplementary documents in Killen *et al.* (2017) and Killen *et al.* (2016), respectively.

The MMR of juvenile blue catfish at 20°C ranged between 173.6 and 430.7 mg O_2_ kg^−1^ h^−1^; the mean MMR of blue catfish (283.4 mg O_2_ kg^−1^ h^−1^) was greater than that of 35.4% of the fish species with published MMR values at 20°C (Fig. 2). SMR ranged between 48.6 and 89.9 mg O_2_ kg^−1^ h^−1^, with the mean SMR (67.0 mg O_2_ kg^−1^ h^−1^) greater than that of 38.4% of the fish species with published SMR values (Fig. 2).

First-order autoregressive covariance structure (ar1) was chosen as the most parsimonious structure for the models describing variation in MMR and FS, suggesting that early measurements of these variables affected subsequent measurements and that the correlations declined exponentially with time (Table 1). The most parsimonious structure for models describing the variation in FL and SMR was the heterogeneous ar1 structure, suggesting that adjacent measurements were more highly correlated than measurements farther apart in time (similar to ar1) but that the variance (and hence, covariances) in FL and SMR differed among measurement periods (different from ar1; Tables 1 and 2). Finally, the spatial power structure was chosen for the models describing variation in K, suggesting that adjacent measurements were more highly correlated than measurements farther apart in time (similar to ar1) but that the number of days between measurements was also important to consider (Table 1).

**Table 1:** Akaike’s information criterion adjusted for small sample size (AICc) for different covariance structures applied to repeated measures mixed-effects models for various response variables

Response	Unstructured	Autoregressive (1)	Heterogeneous autoregressive (1)	Toeplitz	Heterogeneous Toeplitz	Spatial power
FL	452.3	488.5	**450.3**	486.3	454.2	487.3
K	597.9	597.2	597.6	598.8	598	**593.9**
MMR	1120.8	**1104.3**	1110.4	1107.6	1114	1106.9
SMR	776.9	768.8	**766.7**	772.8	770.8	767.4
FS	266.4	**250.9**	257	253.1	259.5	252.5

For each response variable, the most parsimonious model was identified as the model with lowest AICc value, highlighted here in bold.

K indicates Fulton’s body condition.

**Table 2:** Random effects parameter estimates for repeated measures mixed-effects models for FL, K, MMR, SMR and FS

Parameter	Estimate for FL	Estimate for *K*	Estimate for MMR	Estimate for SMR	Estimate for FS
*ρ*	0.80	0.98	0.29	0.22	0.11
*σ* ^2^		0.001	1324.17		0.32
*σ* ^2^_week 4_	1.83			78.57	
*σ* ^2^_week 8_	3.10			32.00	
*σ* ^2^_week 12_	5.30			29.37	
*σ* ^2^_week 16_	14.50			55.87	

*σ*  ^2^ = random unexplained variance; *ρ* = correlation.

Subscripts for variances correspond to specific measurement period; see Table 1 and text for model details.

**Figure 3:**
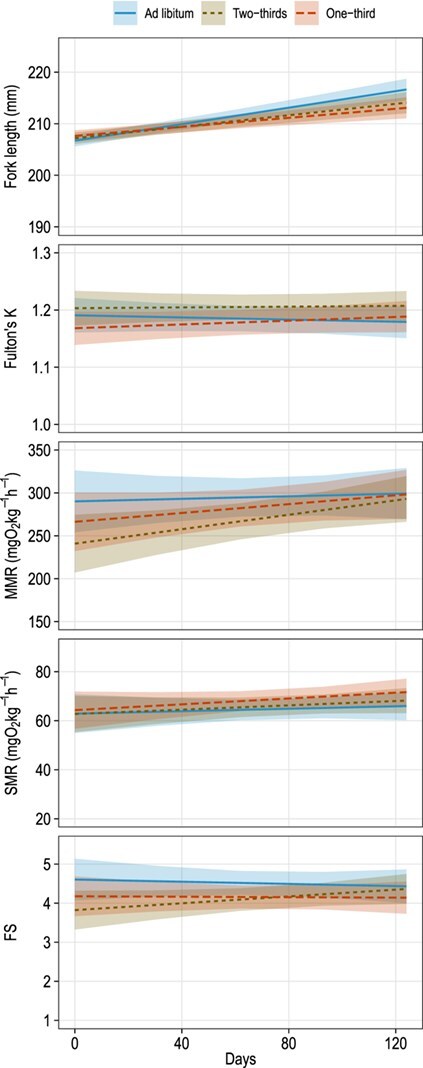
Predicted mean FL, K, MMR, SMR and FS of juvenile blue catfish fed *ad libitum*, two-thirds of *ad libitum* or one-third of *ad libitum* ration size for 124 days. Polygons around each line denote the corresponding 95% confidence bands. Y-axis scales differ among panels.

**Figure 4:**
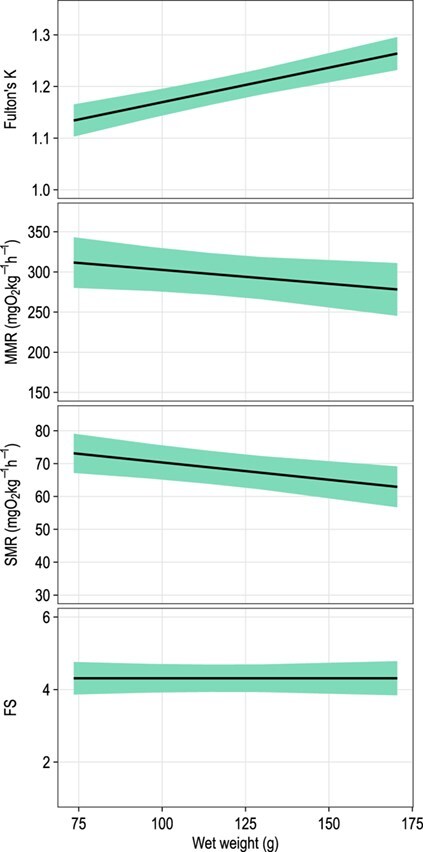
Effect of fish wet weight on predicted mean K, MMR, SMR and FS of juvenile blue catfish. Polygons around each line denote the corresponding 95% confidence bands. Y-axis scales differ among panels.

As expected, ration size had a significant positive effect on the mean growth rate (change in FL per unit time) of juvenile blue catfish (*F*_2, 77.9_ = 4.16; *P* = 0.019; Table 3). Blue catfish that were fed one-third ration size grew slowest (mean = 0.014 mm/d), and those that were fed *ad libitum* grew at significantly faster (mean = 0.080 mm/d) than those fed one-third ration size (*t*_79.8_ = 2.84; *P* = 0.006; Fig. 3). Fish fed two-thirds ration size grew at an intermediate rate (mean = 0.056 mm/d), which was marginally lower than fish fed *ad libitum* (*t*_77.5_ = 1.93; *P* = 0.057), but not different from fish fed one-third ration size (*t*_76.5_ = −0.95; *P* = 0.35). Ration size did not have a significant effect on K, SMR, MMR and FS or on the rate of change in these variables during the experiment (*P* > 0.05; Table 3). All responses were positively affected by their baseline values (i.e. the values of the response before the start of the experiment). For example, fish with a higher baseline FL also had a greater FL at the end of the experiment, regardless of ration size (*F*_1, 32.1_ = 2115.66; *P* < 0.001; Table 3). Finally, the weight of the fish during the trials had a significant positive effect on body condition (*F*_1, 43_ = 43.12; *P* < 0.001), marginal negative effect on MMR (*F*_1, 43.4_ = 3.14; *P* = 0.083) and a significant negative effect on SMR (*F*_1, 38.4_ = 8.83; *P* = 0.005) but no effect on FS (*F*_1, 48.1_ < 0.01; *P* > 0.999; Table 3; Fig. 4).

**Table 3:** Results of the hypotheses tests for the significance of each of the fixed effects on various response variables

Response	Effect	Num DF	Den DF	*F*	*P*
FL	Sex	1	32.4	0.01	0.919
	Ration	2	33.9	0.84	0.439
	Days	1	76.8	136.28	<0.001
	**Baseline FL**	1	32.1	2115.66	**<0.001**
	**Days** $\times$**Ration**	2	77.9	4.16	**0.019**
K	Sex	1	34.1	3.28	0.079
	Ration	2	86.9	1.89	0.158
	Days	1	112	0.15	0.700
	**Baseline K**	1	32.5	98.70	**<0.001**
	Days$\times$Ration	2	116	0.71	0.495
	**Weight**	1	43	43.12	**<0.001**
MMR	Sex	1	38.7	2.18	0.148
	Ration	2	89.9	2.48	0.089
	**Days**	1	103	5.26	**0.024**
	**Baseline MMR**	1	38.5	9.12	**0.005**
	Days$\times$Ration	2	106	0.87	0.422
	Weight	1	43.4	3.14	0.083
SMR	Sex	1	35.1	0.01	0.933
	Ration	2	55.5	0.06	0.942
	Days	1	69	3.06	0.085
	**Baseline SMR**	1	35.1	8.52	**0.006**
	Days$\times$Ration	2	68.1	0.16	0.854
	**Weight**	1	38.4	8.83	**0.005**
FS	Sex	1	44.3	2.60	0.114
	Ration	2	89.9	2.74	0.070
	Days	1	98.6	0.28	0.595
	**Baseline FS**	1	45.2	12.78	**0.001**
	Days$\times$Ration	2	101	1.18	0.310
	Weight	1	48.1	<0.01	>0.999

For each response, the effects that are significant at α = 0.05 are highlighted in bold. However, the main effect is not directly interpretable if it has a significant interaction with another effect; such main effects are not presented in bold.

## Discussion

Blue catfish exhibited relatively low mass-specific metabolic rates in comparison to most fish species (Killen *et al.*, 2016, 2017). A reduction in ration to as low as one-third of *ad libitum* ration size had no negative impacts on the mean body condition and mean metabolic rates of blue catfish, although a small but significant negative effect on mean growth rates was observed. Together, our observations suggest that blue catfish will be able to survive, and subsequently to maintain sustainable populations, in low-food environments.

The mean SMR and MMR of blue catfish were less than those observed in about two-thirds of fish species, likely owing to the benthic lifestyle and feeding strategy of this species. Because of the low energetic investment required to maintain homeostasis and to search for and consume food, many benthic fishes have evolved to have low metabolic demands as characterized by low SMR and MMR (Killen *et al.*, 2016, 2017). Low metabolic rates allow blue catfish to tolerate environments (or time periods) with low or patchy resources, but at the expense of slower growth rates (Killen *et al.*, 2016). Pelagic and benthopelagic fishes common in Chesapeake Bay have higher metabolic rates than blue catfish (e.g. Freadman, 1981; Marcek *et al.*, 2019). Based on the resource-ratio hypothesis (Tilman, 1982), we conclude that blue catfish will have a competitive advantage in low-food habitats. In addition, blue catfish are generalist omnivores that have a particularly high diet breadth compared with other estuarine fishes (Schmitt *et al.*, 2019). The competitive advantages conferred by these characteristics suggest that blue catfish are likely to outcompete native species resulting in declines in abundance of native species via suppression or displacement. Indeed, observed declines in relative abundance of the congeneric native white catfish (*Ameiurus catus*) in Chesapeake Bay subestuaries during the past few decades may have resulted from exploitative competition with blue catfish (Fabrizio *et al.*, n.d.; Schloesser *et al.*, 2011).

Blue catfish grew in length at all ration treatments, consistent with life-history trade-off mechanisms: when food is limited, juvenile fish may allocate more energy to growth as measured by an increase in length (Garvey and Marschall, 2003). Length increases are prioritized over gonadal development or storage (increases in weight) because growth that leads to a larger eventual body size allows for greater future reproductive output (Stearns, 1992). If, instead, a fish invested energy towards reproduction when it was young, its growth rate would decline, thereby permanently reducing the likelihood of a high lifetime reproductive output.

The mean body condition of blue catfish was generally stable over the 4-month experimental period and indistinguishable among fish from the different ration treatments. This could be because the increase in food conversion efficiency at lower ration sizes (Abbas and Siddiqui, 2009; Liu and Fu, 2017) may result in relatively stable body weights and, hence, stable body condition when ration size is reduced. Also, the loss of body mass at reduced ration sizes is often partially offset by an increase in the proportion of water, resulting in relatively low declines in overall wet weight (Brett *et al.*, 1969; Abbas and Siddiqui, 2009; McCue, 2010).

Reduced ration size did not have a significant effect on mean SMR or MMR, and consequently, FS did not change with ration level either. Previous research shows that even after accounting for the energy costs associated with the burst of protein synthesis following feeding (i.e. specific dynamic action), well-fed fish tend to have a higher SMR compared with starved fish (Van Leeuwen *et al.*, 2011, 2012; Auer *et al.*, 2016; Liu and Fu, 2017). This is because low rates of feeding induce plastic decreases in the size of the digestive tract resulting in lower maintenance costs (i.e. lower SMR; Shoemaker *et al.*, 2003; Abbas and Siddiqui, 2009; Armstrong and Bond, 2013). Fishes with low SMR, however, generally lack this plastic ability to regulate metabolism. For example, salamanderfish (*Lepidogalaxias salamandroides*) (mean SMR = 50 mL O_2_ kg^−1^ h^−1^) and traíra (*Hoplias malabaricus*) (mean SMR = 42 mL O_2_ kg^−1^ h^−1^), two species with low metabolic rates under *ad libitum* or normal feeding conditions, do not demonstrate declines in SMR despite 40 and 180 days of starvation, respectively (Pusey, 1986; Rios *et al.*, 2002). For such species, further reduction in metabolic expenditures might compromise the ability to maintain physiological homeostasis (Pusey, 1986; Rios *et al.*, 2002). This may explain why we did not observe declines in SMR in blue catfish even at the lowest ration size. Few researchers have studied the effects of food reduction on MMR and those that have suggest that the nutritional state of a fish may not greatly influence its MMR (Van Leeuwen *et al.*, 2011; Auer *et al.*, 2016). Finally, change in FS depends on the response of SMR and MMR. Because we did not observe differences in SMR and MMR during the experimental period, it is not surprising that FS did not change with ration level. Overall, the metabolism of invasive blue catfish in established habitats does not seem to change in response to food availability.

Food restriction may affect other aspects of blue catfish biology that we did not measure. During starvation, fishes use endogenous reserves to maintain homeostasis, grow and even reproduce (Kooijman, 2010; McBride *et al.*, 2015), thereby changing their energy density and body composition. For example, the total energy content of largemouth bass (*Micropterus salmoides*) declines under food restriction (Garvey *et al.*, 1998) and starved sockeye salmon (*Oncorhynchus nerka*) have a lower proportion of fat than well-fed fish (Brett *et al.*, 1969). Similar results were reported for channel catfish (*I. punctatus*) under food restriction (Shoemaker *et al.*, 2003). Differential utilization of metabolic compounds (i.e. lipids versus proteins) during starvation can also affect the weight and body condition of a fish (McCue, 2010). Because lipids are more energy-dense (9 calories/g) than proteins (4 calories/g), prioritized use of reserve lipids would result in lower weight loss. Studies on changes in body composition and hormonal levels during starvation would be useful in uncovering such patterns in blue catfish.

Finally, our results do not account for the developmental and environmental histories of fish. Recent studies suggest that pre-exposure to starvation events often induces adaptive responses such as reduced rates of mass loss, reduced metabolic rates and lower costs of digestion during subsequent starvation events (reviewed in McCue *et al.*, 2017). In tidal tributaries of the Chesapeake Bay and, more specifically, in the James River from where the experimental fish were collected, the densities of the fish are high (Fabrizio *et al.*, n.d., 2018; Nepal and Fabrizio 2020a), and thus fish used in our studies may have experienced starvation prior to capture. Importantly, these observations suggest that metabolic rates and the response of blue catfish to reduced food availability have changed since the introduction of the species into the region. Further, fish on the leading edge of the range or in newly invaded rivers may have higher metabolic rates, which can confer a competitive advantage by increasing aggressiveness and growth rates (Myles-Gonzalez *et al.*, 2015). It may also, in part, explain why blue catfish growth rates were higher during the early years of invasion (Nepal and Fabrizio, 2020a; Nepal *et al.*, 2020).

The lack of negative impacts of reduced rations could also result from ration sizes that were too large, regardless of treatment level. If our *ad libitum* ration size (roughly 3.5% of fish biomass per day) was much higher than the actual *ad libitum* ration size, then the lower ration sizes would have been sufficient to avoid negative impacts on condition and metabolic rates. Indeed, we noted uneaten food in several aquaria at *ad libitum* ration level, although the frequency of such occurrences was lower in the reduced ration treatments. Studies support that the ration size used in our experiment reflected true feeding rates of juvenile blue catfish. Blue catfish from the same population as our study animals (tidal James River) have maximum daily consumption rates of ≈9% body mass per day when starved for 72 hours; field estimates of daily ration range between 2.2% and 5.2% body mass per day (Schmitt, 2018). Likewise, channel catfish (a blue catfish congener) consumes ~3.4% of fish biomass per day (Green and Rawles, 2010). It seems unlikely, therefore, that our *ad libitum* ration size was too high. The reduced ration sizes were higher than maintenance ration as demonstrated by fish growth at these ration sizes. Li *et al.* (2004) report maintenance ration of approximately 1/7th (i.e. 14.3%) of *ad libitum* ration size for catfish (unknown North American species) fed commercial catfish feed. In close agreement to Li *et al.*’s findings, a full life-cycle bioenergetics model for blue catfish from the James River suggests that ration sizes <17.3% of the maximum would prevent a fish from growing to maturity (Nepal, 2020). The reduced ration sizes we used, therefore, seem to be high enough to allow growth, albeit at a slower rate. This is particularly relevant as the energy density of the catfish pellets we used was likely greater than that of the prey items consumed by blue catfish in the wild. Energy density and proximate composition of the food can affect the energy absorption efficiency (Targett and Targett, 1990) and energy allocation in fish (Garvey *et al.*, 1998).

Our findings highlight the concern about further range expansion and potential negative impacts of blue catfish in non-native environments such as the Chesapeake Bay and Atlantic slope rivers of the Eastern USA. Range expansion and impacts of blue catfish in Chesapeake Bay may not be hindered by the estuarine salinity gradient (Nepal and Fabrizio, 2019) or climate warming (Nepal and Fabrizio, 2020a, b). Here, we report that blue catfish have low energetic demands and are able to maintain positive growth in low-food environments. Blue catfish may withstand food limitation for at least 4 months without reductions in SMR, MMR or FS and thus without diminution of their ability to increase metabolic rate following feeding or bouts of exhaustive activity. In aggregate, our results show that blue catfish may have low somatic maintenance and high reserve capacity (*sensu*  Kooijman, 2010) and that severe and long periods (>4 months) of food limitation are needed to hamper growth and reproduction. We contend that such conditions are not likely commonly encountered by blue catfish in most estuarine habitats today, particularly given their omnivorous feeding behaviour. Blue catfish, therefore, have the potential to disperse to, and establish in, the areas of low prey density. Importantly, blue catfish could also use low-food habitats as a ‘stepping-stone’ to disperse to more suitable and energetically rich habitats. Metabolic characteristics of blue catfish, therefore, seem conducive to range expansion and establishment into novel habitats with low or patchy food availability.

Experimental approaches based on physiological characteristics provide mechanistic understanding of the distribution of a species (Kooijman, 2010; Horodysky *et al.*, 2015). Because physiology directly links environmental conditions with fitness and behaviour of an individual, inferences based on physiological characteristics are highly robust to extrapolation to novel habitats (Horodysky *et al.*, 2015). Physiology-based approaches, therefore, are increasingly being prioritized in research on invasive species (Augustine *et al.*, 2014; Myles-Gonzalez *et al.*, 2015; Meyerson *et al.*, 2019; Hasenei *et al.*, 2020). In this study, we used macro-physiological and life-history traits to investigate the invasion potential of blue catfish. Results suggest that resource managers and conservationists should be concerned about the potential for blue catfish to continue their range expansion and establishment in mid-Atlantic estuaries and to negatively impact native resources of economic, cultural or conservation value. It appears that food availability will not be a limiting factor in their potential range expansion. This non-native species may alter estuarine ecosystem structure and function and potentially result in the loss of ecosystem services. In particular, with rising temperatures, feeding rates of blue catfish are likely to increase (Brett *et al.*, 1969), exacerbating negative impacts on native organisms. Resource managers should, therefore, strive to prevent further expansion of blue catfish into novel areas through vigilant monitoring and targeted removals.

## Funding

This work was supported by the Virginia Sea Grant (Grant number 71856W), the Office of Academic Studies at the Virginia Institute of Marine Science, the Virginia Marine Resources Commission and the United States Fish and Wildlife Service.
